# Analysis of Kif5b Expression during Mouse Kidney Development

**DOI:** 10.1371/journal.pone.0126002

**Published:** 2015-04-17

**Authors:** Ju Cui, Xiuling Li, Zhigang Duan, Wenqian Xue, Zai Wang, Song Lu, Raozhou Lin, Mengfei Liu, Guixia Zhu, Jian-Dong Huang

**Affiliations:** 1 The Key Laboratory of Geriatrics, Beijing Hospital & Beijing Institute of Geriatrics, Ministry of Health, Beijing, China; 2 Department of Biochemistry, LKS Faculty of Medicine, The University of Hong Kong, Hong Kong SAR, China; 3 Department of Anatomy, LKS Faculty of Medicine, The University of Hong Kong, Hong Kong SAR, China; 4 Institute of Clinical Medical Sciences, China-Japan Friendship Hospital, Beijing, China; 5 The Centre for Synthetic Biology Engineering Research, Shenzhen Institutes of Advanced Technology, Shenzhen, PR China; UCL Institute of Child Health, UNITED KINGDOM

## Abstract

Recent studies showed that kidney-specific inactivation of *Kif3a* produces kidney cysts and renal failure, suggesting that kinesin-mediated intracellular transportation is important for the establishement and maintenance of renal epithelial cell polarity and normal nephron functions. Kif5b, one of the most conserved kinesin heavy chain, is the mouse homologue of the human ubiquitous Kinesin Heavy Chain (uKHC). In order to elucidate the role of Kif5b in kidney development and function, it is essential to establish its expression profile within the organ. Therefore, in this study, we examined the expression pattern of Kif5b in mouse kidney. Kidneys from embryonic (E) 12.5-, 16.5-dpc (days post coitus) mouse fetuses, from postnatal (P) day 0, 10, 20 pups and from adult mice were collected. The distribution of Kif5b was analyzed by immunostaining. The possible involvement of Kif5b in kidney development was investigated in conditional mutant mice by using a Cre-LoxP strategy. This study showed that the distribution of Kif5b displayed spatiotemporal changes during postnatal kidney development. In kidneys of new born mice, Kif5b was strongly expressed in all developing tubules and in the ureteric bud, but not in the glomerulus or in other early-developing structures, such as the cap mesenchyme, the comma-shaped body, and the S-shaped body. In kidneys of postnatal day 20 or of older mice, however, Kif5b was localized selectively in the basolateral domain of epithelial cells of the thick ascending loop of Henle, as well as of the distal convoluted tubule, with little expression being observed in the proximal tubule or in the collecting duct. Conditional knock-down of Kif5b in mouse kidney did not result in detectable morphological defects, but it did lead to a decrease in cell proliferation rate and also to a mislocalization of Na^+^/K^+^/-ATPase, indicating that although Kif5b is non-essential for kidney morphogenesis, it is important for nephron maturation.

## Introduction

Kidney diseases are common in human populations worldwide. The normal activities of the kidney include the excretion of metabolic waste and fluid in the form of urine, the homeostatic regulation of electrolytic and acid-base balance, and endocrine functions. The structural and functional unit within the kidney is the nephron, which comprises the glomerulus, the proximal tubule, the loop of Henle and the distal convoluted tubule [1].

Kidney morphogenesis in the mouse has been extensively studied and reviewed [2, 3]. Organogenesis in the mammalian kidney consists of three stages: pronephros, mesonephros and metanephros. The metanephros stage is initiated at E10.5-E11, starting from the reciprocal interaction between the Wolffian duct and the metanephric mesenchyme (MM). The ureteric bud invades the MM, and promotes the induction of cap mesenchyme. Subsequently, the cap mesenchyme proceeds through a series of morphological stages: pre-tubular aggregates, renal vesicles, comma-shaped body, S-shaped body and capillary loop nephron, it then differentiates further to give rise to glomerulus, proximal tubule, loop of Henle and distal tubule. The distal tubule connects to the ureteric bud, which becomes transformed into collecting duct. Nephrogenesis ceases at 3 days after birth, and from this time onwards no additional nephrons are initiated, but the induced immature nephrons continue to proliferate and differentiate until they become mature ones [4]. Around postnatal day 21, most, if not all, of the nephrons become mature and functional, as shown by the observation that the proliferation of renal tubular cells is very clearly diminished at postnatal day 21 compared to the new born stage [5].

Although the morphological changes that occur during kidney organogenesis have been elucidated, the underlying molecular mechanisms remain largely unknown. Kidney-specific knockout of Kif3a in mouse inhibits renal ciliogenesis and results in polycystic kidney disease (PKD) [5, 6], highlighting the importance of intracellular transportation in kidney development and function.

Kinesin-1 (conventional kinesin) is a microtubule plus-end-directed motor protein, comprising two heavy chains (KHCs) and two light chains (KLCs) [7]. Three KHC isoforms have been identified in mouse, denoted Kif5a, Kif5b and Kif5c. Kif5a and Kif5c are neuron-specific, whereas Kif5b could be detected in all tissues by Western blot analysis [8]. This expression pattern suggests that Kif5b is involved in many more intracellular activities than Kif5a/c; this is further supported by the fact that conventional *Kif5b* knockout mice die at E8.5–10.5 [9], whereas *Kif5a* null mice are postnatal-lethal [10] and *Kif5c* null mice, by contrast, are viable and fertile [8].

Based on biochemical studies, Kif5b has been revealed to mediate the transportation of a diversity of cellular compartments, such as organelles, vesicles, proteins and mRNAs (reviewed by [11]). Although significant progress has been made in characterizing the functions of Kif5b in neuronal tissues, corresponding information regarding its functions in non-neuronal tissues remains fragmentary; so far, a small number of interacting cargoes have been identified and some cellular functions have been suggested [12–17]. In polarized MDCK cells, post-Golgi transport of p75, an apical protein in epithelial cells, was found to be mediated by Kif5b [18], and in non-polarized MDCK cells, trafficking of p75 was carried out by Kinesin-3 family members (KIF1A and KIF1Bβ) [19]. Although Kif5b selectively transports p75 to the apical membrane in polarized MDCK cells, the possibility cannot be excluded that Kif5b may be involved in basolateral transportation in epithelial cells. Kinesin light chain-2 of Kinesin-1 (KLC2) participates in the transportation of basolaterally localized Na^+^-K^+^ ATPase-containing vesicles in alveolar epithelial cells [20]. In human oral squamous carcinoma SCC-9 cells, Kif5b interacts with desmosomal cadherin Dsg2 to regulate the normal Dsg2 accumulation in desmosomes [21]. Furthermore, in Hela cells, p120 catenin (p120) forms a complex with kinesin heavy chain (Kif5b) to facilitate the transport of N-cadherin-catenin complexes to adhesion junctions [16], indicating a possible involvement of Kif5b in E-cadherin transportation in epithelial cells. Finally, in colonic epithelial cells, Kif5b is localized at intact and internalized apical junctions and can mediate the disassembly/internalization of adhesion junctions (E-cadherin) and tight junctions (occludin) upon Ca^2+^depletion [22]. Identification of these cargoes in a range of epithelial cells therefore suggests that Kif5b may play an important role in renal tubular epithelial cell polarity and function.

Against this background, and as a first step in obtaining clues to the functions of Kif5b in kidney development and function *in vivo*, we have in this study examined the spatiotemporal expression patterns of Kif5b in mouse kidney.

## Materials and Methods

### Animals

C57BL/6N, ROSA26 reporter line, *Kif5b* ^*+/-*^, *Kif5b* ^*fl/fl*^ and *Pax2-Cre* mice were used in this study. Generation of *Kif5b* ^*+/-*^:*Pax2-Cre* mice has been described elsewhere [23]. The day on which the cervical mucus plug was observed was designated as embryonic day 0.5 (E0.5) and the day on which birth occurred was designated as postnatal day 0 (P0). For each time point, three mice were obtained from separate litters. Mouse experimentation was carried out in strict accordance with the recommendations in the guide for the Care and Use of Laboratory Animals of the United States National Institutes of Health. The protocol was approved by the Committee on the Use of Live Animals in Teaching and Research (CULATR) at The University of Hong Kong (Permit Number: 2540–11). All surgery was performed under sodium pentobarbital anesthesia, and all efforts were made to minimize animal suffering. In total, more than ten litters of mutant and control mice or embryos were examined in this study.

### Total protein extraction and Western blot

Fresh kidney tissues were washed with cold PBS and lysed in RIPA buffer (50 mM Tris-Cl (pH7.4), 150 mM NaCl, 1% NP-40, 0.5% Sodium Deoxycholate, 1mM EDTA, 0.1% SDS, 0.01% Sodium Azide). The protein concentrations of lysates were determined using a BCA Protein Assay Kit (Thermo). Equal amounts of protein samples were loaded onto SDS-PAGE gels for electrophoresis and subsequent Western blot analysis. The Kif5b-specific antibody used has been described elsewhere [24]. The blots were incubated with anti-Kif5b primary antibody (1:2000, against synthesized peptide FDKEKANLEAFTADKDIA), anti-Kif5a (1:100, against synthesized peptide NGNATDINDNRSDLPC), anti-Kif5c (1:100, against synthesized peptide SAKDQKSLEPC) (S1 Fig) and anti-actin primary antibody (1:3000, Sigma) at 4°C overnight, followed by incubation with HRP-(horseradish peroxidase)-conjugated secondary antibodies at room temperature for 1 hour. An enhanced chemiluminescence kit (Pierce) was used for detection of the immunoreactive bands.

### Paraffin sections and immunostaining

Kidneys were dissected from anaesthetized mice either directly, or after transcardial perfusion with PBS. Tissues were fixed with 4% paraformaldehyde (PFA) in phosphate-buffer saline (PBS) at 4°C overnight. The tissues were dehydrated in a series of ethanol solutions of increasing concentration, and then cleared using xylene or toluene and embedded in wax. The wax blocks were sectioned at 7 μm using a rotary microtome (Leica). The wax sections were floated in a water bath at 42°C to allow full expansion, and were then mounted on glass slides. The slides were air-dried, heated in a 65°C oven for 1 hour and stored at room temperature.

For immunohistochemical analysis, rehydrated paraffin sections were briefly rinsed with PBS and blocked with Peroxidase-Blocking Reagent (Dako) for 10 min, and then incubated with blocking solution (5% [w/v] goat serum, 3% [w/v] BSA [bovine serum albumin] and 0.5% [v/v] Triton X-100 in PBS) for 1 hour at room temperature, followed by incubation with rabbit anti-Kif5b antibody (1:200) at 4°C overnight. After being washed with TBST (Tris-buffered saline with Tween-20), the sections were incubated with HRP-conjugated anti-rabbit secondary antibody at room temperature for 1 hour. Staining for Kif5b was undertaken using liquid DAB (3,3'-diaminobenzidine) and the substrate-chromogen solution from the EnVision+ System-HRP (DAB) kit (Dako), with hematoxylin counter-staining. Specimens on slides were observed under a light microscope (Carl Zeiss) and photographed using a Microphoto-FX microscope (Nikon) attached to a digital camera (Nikon).

For immunofluorescent staining, sections were permeabilized and blocked using blocking solution (5% [w/v] donkey serum, 3% [w/v] BSA and 0.5% [v/v] Triton X-100 in PBS), followed by incubation with primary antibodies diluted in blocking solution overnight at 4°C, comprising rabbit anti-Kif5b antibody (1:200), rabbit anti-NCC antibody (1:200, Chemicon), goat anti-THP antibody (1:100, Santa Cruz) and goat anti-AQP2 (1:100, Santa Cruz), and mouse anti- Na^+^/K^+^-ATPase antibody (1:100, Millipore). Visualization of target proteins was realized by incubation with Alexa Fluor 488 (Invitrogen)-, Cy3- or rhodamine- conjugated secondary antibodies (Jackson ImmunoResearch Laboratories) for 1 hour at room temperature and mounting with *SlowFade* Gold antifade reagent with DAPI (4',6'-diamidino-2-phenylindole). For labeling proximal tubules and collecting ducts, sections were stained using biotinylated *Lotus tetragonolobus* lectin (1:200, Vector Laboratory) and biotinylated *Dolichos biflorus* agglutinin (1:200, Vector Laboratory) respectively, and detected using fluorescein Avidinin D (1:400, Vector Laboratory). Fluorescence was detected by fluorescence microscopy (Zeiss Axiophot 2) and Carl Zeiss LSM 700 confocal microscope.

### Total RNA extraction and quantitative PCR

Total RNA was isolated from tissues using Trizol reagent (Invitrogen). First-strand cDNA was synthesized using Superscript III reverse transcriptase (Invitrogen). Quantification of mRNA levels was performed using SYBR Premix Ex Taq (Takara). The reference gene chosen was *18S* ribosomal RNA. The sequences of the primers are listed in S1 Table.

### Quantitation of cell proliferation in kidney

Cell proliferation in kidneys was determined by 5-bromo-2’-deoxyuridine (BrdU) incorporation using a BrdU staining kit (ZYMED Laboratory). Pregnant female mice received an intraperitoneal injection of BrdU (100 μg/g body weight) 4 hours prior to being killed. Identification of BrdU-positive cells was performed by immunostaining as described previously [24].

### Statistical analysis

Sigma Stat (Systat Software) was used to analyze the data which were expressed as the mean ± S.D. Comparisons between two mean values were performed by independent samples *t*-test. For multiple comparisons among different groups of data, significant difference were determined by one-way analysis of variance (ANOVA) followed by Tukey’s test. *P*<0.05 was considered to be statistically significant.

## Results and Discussion

### Kif5b is widely expressed in tubular cells in the neonatal mouse kidney

Although Kif5b is widely expressed in mouse tissues [8], it exhibits various expression levels in different cell types within a specific organ. In the nervous system, Kif5b was found to be highly expressed in olfactory sensory neurons [8], whereas in the pancreas Kif5b was predominantly localized in islets, with little expression in exocrine acinar cells in adult mice [24]. It is currently poorly documented whether the distribution of Kif5b varies among different cell types in the kidney.

**Fig 1:**
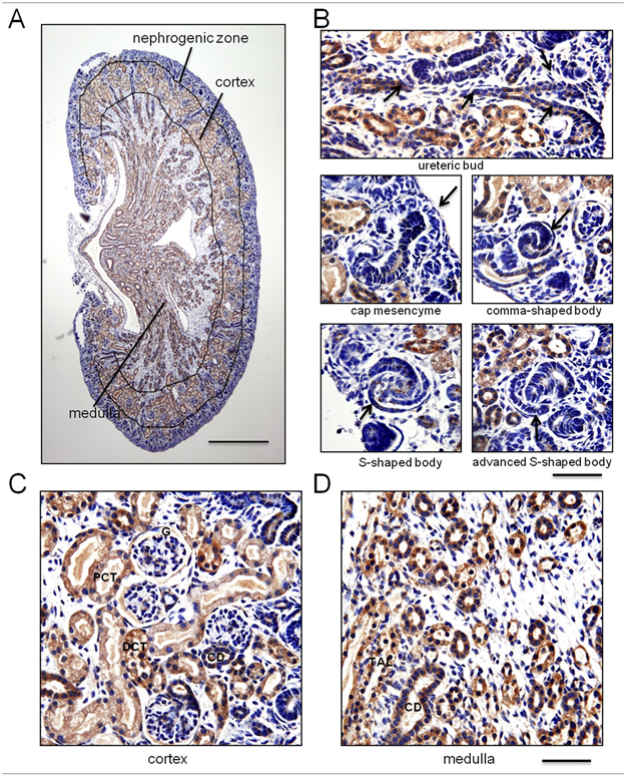
Expression pattern of Kif5b in newborn mouse kidney. (A) Overall view of Kif5b expression in the kidney: strong expression in tubular structures in both cortex and medulla, and low levels of expression in the nephrogenic zone. (B) In the nephrogenic zone, Kif5b was expressed in the ureteric bud, but exhibited a very low expression in the cap mesenchyme, comma-shaped body, S-shaped body and advanced S-shaped body. (C, D) Kif5b was widely expressed in the renal tubules of the cortex and the medulla, but expression in glomeruli and in interstitial cells was quite rare. G, glomerulus; PCT, proximal convoluted tubules; TAL, thick ascending limbs of loops of Henle; DCT, distal convoluted tubule; CD, collecting duct. Images are representative of kidney tissue sections from five mice. Scale bar: (A) 500 μm, (B-D) 50 μm.

To characterize Kif5b expression in the kidney, we analyzed the distribution of Kif5b in neonatal kidneys. In the newborn mouse, the kidney can be divided into three parts: the outermost part is the nephrogenic zone where nephrogenesis occurs, and the inner parts are the cortex and the medulla, respectively (Fig 1A). Kidney cortex is composed of glomeruli and renal tubules, while the medulla comprises only tubules. The interstitium is filled among the parenchyma in the whole kidney.

Based on immunohistochemical analysis, Kif5b was found to be localized mainly in the renal tubules of the cortex and medulla, with little expression in the nephrogenic zone or glomeruli of the cortex (Fig 1A). The positive staining was absent after pre-incubating the anti-Kif5b antibody with the 18-aa synthesized peptides (S1C Fig). In the nephrogenic zone, Kif5b was expressed prominently in the ureteric bud, but it exhibited a very low level of expression in the cap mesenchyme, the comma-shaped bodies, the S-shaped bodies and the advanced S-shaped bodies (Fig 1B). This indicates that Kif5b is expressed more strongly in structures that are of Wolffian duct origin than in MM-derived early nephron structures. We next examined the expression of Kif5b in the MM-derived late nephron structures: the glomeruli and renal tubules in the cortex and medulla. We found that Kif5b was widely expressed in the renal tubules of both cortex and medulla, such as the proximal convoluted tubules (PCT), the thick ascending limbs of the loops of Henle (TAL), the distal convoluted tubules (DCT) and the collecting ducts (CD), but the expression in glomeruli was quite rare (Fig 1C and 1D). Expression of Kif5b in the interstitial cells was also hardly detectable (Fig 1B–1D).

### The expression pattern of Kif5b changes with kidney development

**Fig 2:**
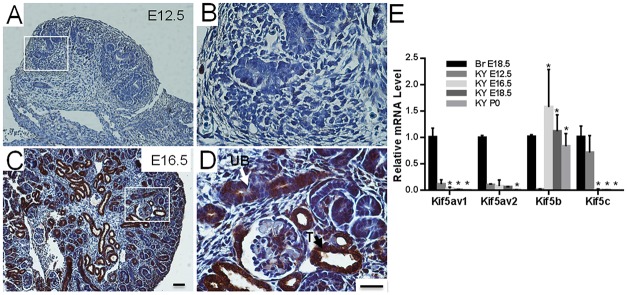
Embryonic developmental expression of Kif5b in mouse kidney. (A-D) Immunolocalization of Kif5b protein in the fetal mouse kidney at embryonic days (E) 12.5, 16.5. (A, B) Kif5b was not detectable in the E12.5 fetal kidney. (C, D) At E16.5, Kif5b was highly expressed in the ureteric bud (UB) and tubules (T). Images are representative of kidney tissue sections from three mice. (E) Quantitative real-time RT-PCR analysis showing the expression of Kif5 members: Kif5a, Kif5b and Kif5c mRNAs during kidney development. Br E18.5, brain of E18.5 embryo; KY E12.5, kidney of E12.5 embryo. mRNAs of brain tissues from E18.5 embryos were used as positive controls in the real-time PCR analysis. Bars represent the means ± S.D. (n = 3). One-way ANOVA was performed to compare specific gene expression levels in the kidneys between different stages. * *P*<0.05 versus KY E12.5. Scale bar = 25 μm.

To characterize the spatial dynamics of Kif5b expression during kidney development, we examined the expression pattern of Kif5b in E12.5, E16.5, P0, P10, P20 and adult kidneys. Expression of Kif5b was not detectable in the kidneys of E12.5 embryos (Fig 2A and 2B); however, in E16.5 kidneys, Kif5b was highly expressed in the ureteric bud and tubular structures (Fig 2C and 2D). The quantitative levels of Kif5b mRNA during embryonic kidney development were determined by real-time reverse transcription PCR (qRT-PCR) (Fig 2E). The Kif5b mRNA was not detected in the early embryonic kidney (E12.5), but its level increased significantly in the kidneys of E16.5 embryos and thereafter. Although Kif5a and Kif5b were reported to be neuronal specific [8], both of them could be detected in E12.5 kidneys. The expression levels of Kif5a and Kif5c declined significantly during later kidney development, and could not be detected at all in the kidneys of newborn mice (Fig 2E and S1D Fig)

**Fig 3:**
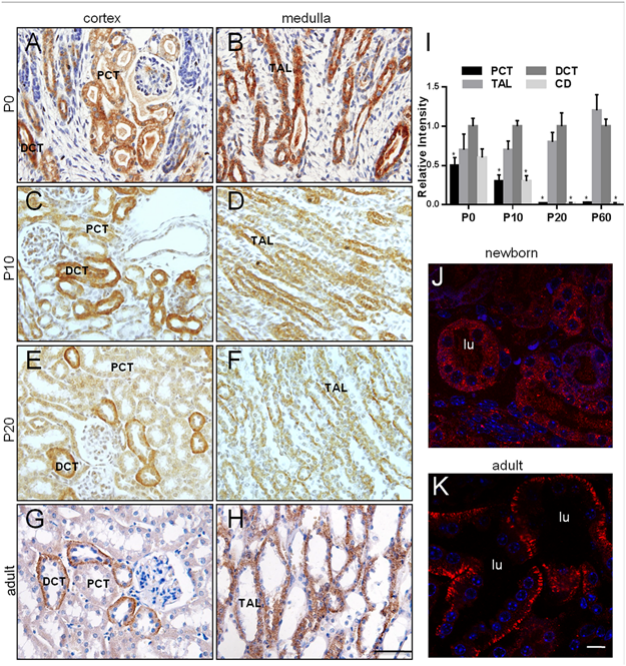
Spatiotemporal expression pattern of Kif5b in mouse kidney. (A, B) Kif5b was widely distributed inside renal tubular epithelial cells of newborn mice. (C-H) Kif5b was gradually restricted to epithelial cells in DCT and TAL during postnatal kidney development. (I) Quantitative analysis of the relative optical density values for cells in renal tubules. Kidney sections were analyzed using ImageJ software to determine the mean optical density values per cell of positive-labeled tubules in the kidney. The relative mean optical densities of cells in the tubular structures compared with that in the DCT were calculated. Bars represent the means ± S.D. (n = 10 for each tubular structure from three mice were measured and analyzed). One-way ANOVA was performed to compare relative intensity levels of different tubular structures at indicated stages. * *P*<0.05 versus DCT. (J) Kif5b was evenly distributed in the cytoplasm of renal epithelial cells in newborn mice. (K) Kif5b was asymmetrically distributed in the basolateral domain in renal tubular cells of adult mice. Lu, lumen; PCT, proximal convoluted tubules; TAL, thick ascending limbs of loops of Henle; DCT, distal convoluted tubule; CD, collecting duct. Images are representative of kidney tissue sections from three mice. Scale bar: (A-H) 50 μm, (I, J) 10 μm.

In both the cortex and the medulla, the spatial expression of Kif5b changed with development in the postnatal kidney in two aspects. First, Kif5b expression gradually became restricted to DCT and TAL (Fig 3A–3I). This alteration became obvious at P20 (Fig 3E, 3F and 3I), at which stage the renal tubules probably became functionally mature. Secondly, the subcellular localization of Kif5b changed during development. In the newborn mouse, Kif5b was evenly distributed in the cytoplasm of renal epithelial cells (Fig 3A, 3B and 3J), whereas it was asymmetrically distributed in the basolateral domain in renal tubular cells of adult mice (Fig 3G, 3H and 3K). Although Kif5b was preferentially expressed in some tubules during postnatal kidney development, its total expression level in the whole kidney remained unchanged during this process (S1E Fig).

The presence of Kif5b in the tubules of mouse kidney indicates that Kif5b may be involved in a re-absorption and/or secretion process specific to renal tubules. Furthermore, dynamic spatial changes either intracellularly or along the length of differentiating renal tubules (Fig 3) indicate a requirement of Kif5b during kidney postnatal development.

### Kif5b is selectively expressed in the basolateral domain of cells in the thick ascending limbs (TAL) and distal convoluted tubules (DCT) in the adult mouse

**Fig 4:**
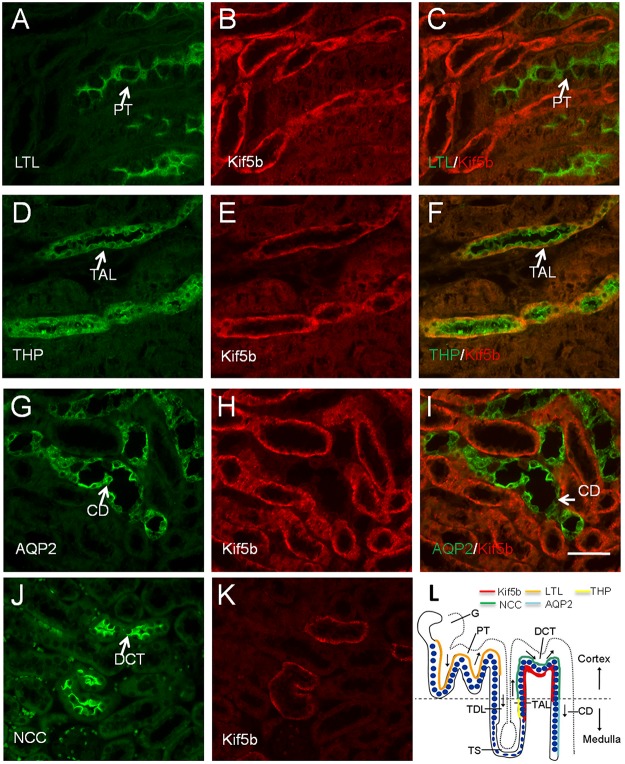
Immunolocalization of Kif5b in mouse kidney PT (A-C), TAL (D-F), CD (G-I) and DCT (J-K), using antibodies against specific markers for each segment. Kif5b was expressed in TAL (THP-positive tubules) and DCT (NCC-positive tubules), but not in PT (LTL-positive tubules) or CD (AQP2-positive tubules). (L) The diagram shows the selective and asymmetric expression pattern of Kif5b in renal tubules of adult mice. Red, Kif5b; orange, LTL; yellow, THP; green, NCC; light blue, AQP2; blue, nucleus. Only cells from one side of the tubules are presented and the other side is represented by a dotted line. Arrows represent the direction of urinary flow. Cortex and medulla are separated by a dashed line. G, glomerulus; PT, proximal tubule; TDL, thick descending limbs of Henle; TS, thin segment; TAL, thick ascending limbs of Henle; DCT: distal convoluted tubule; CD, collecting duct. Images are representative of kidney tissue sections from three mice. Scale bar = 50 μm.

To further confirm the nephron segments in which Kif5b was expressed in adult mice, kidney paraffin sections were double labelled for Kif5b and for markers of specific nephron segments (Fig 4). *Lotus tetragonolobus* agglutinin (LTL) is a lectin that specifically stains proximal tubules (green) [6]. Double immunostaining showed that Kif5b was not expressed in the proximal tubule (PT). Aquaporin-2 (AQP2) is localized mainly at the apical cell membranes of the principal cells of the CD of the kidney [25]. Lack of Kif5b expression in AQP2-positive tubules revealed that Kif5b was not expressed in the CD. Tamm-Horsfall protein, a GPI-anchored glycoprotein (THP), is found to be produced by the TAL in mammalian kidney [6]. Here, positive staining of Kif5b and THP in the same renal tubules suggested that Kif5b was expressed in the TAL. Finally, the Na-Cl cotransporter (NCC) is specifically localized at the apical surface of the DCT. Since antibodies against NCC and Kif5b were both raised in rabbit, we stained these two proteins separately in two consecutive tissue sections. Kif5b and NCC were found to be expressed in the same tubular structures, indicating that Kif5b was expressed in the DCT.

The selective and asymmetric distribution of motor protein in mouse renal epithelial cells is not unique to Kif5b. KIFC3, a microtubule minus-end-directed motor, was reported to be specifically localized in distal tubules and loops of Henle, with strong staining in the apical area of the epithelial cells within these two nephron segments [26]. KIFC3 was also reported to transport annexin XIIIb-associated vesicles to the apical membrane, and this therefore implied that the plus-end-directed Kif5b was involved in basolateral transportation in epithelial cells in TAL and DCT, a process that may be essential for the normal physiological functions unique to these nephron segments such as transepithelial electrolyte transportation and regulation of urinary concentration[27].

**Fig 5:**
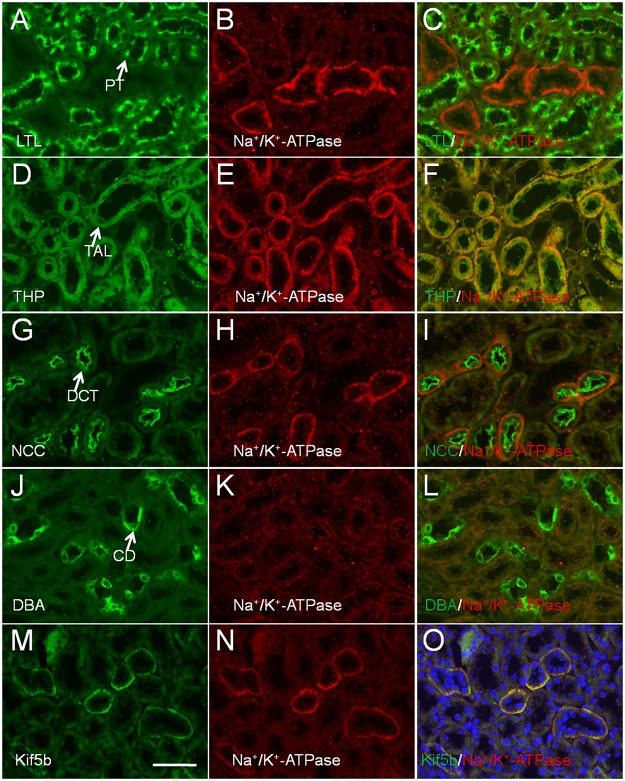
Immunolocalization of Na^+^/K^+^-ATPase in mouse kidney PT (A-C), TAL (D-F), DCT (G-I) and CD (J-L) using antibodies against specific markers for each segment. Kif5b was expressed in TAL (THP-positive tubules) and DCT (NCC-positive tubules), but not in PT (LTL-positive tubules) or CD (DBA-positive tubules). (M-O) Co-localization of Kif5b and Na^+^/K^+^-ATPase in renal tubules of adult mice. PT, proximal tubule; TAL, thick ascending limbs of Henle; DCT: distal convoluted tubule; CD, collecting duct. Images are representative of kidney tissue sections from three mice. Scale bar = 50 μm.

Tubular cells in the TAL and the DCT are rich in mitochondria [28] and have a high density of Na^+^/K^+^-ATPase [29], which is essential for energy-demanding active intracellular transportation [30, 31]. It was reported that Kif5b forms functional Kinesin-1 with Kinesin light chain-2 to regulate the transportation of the Na^+^/K^+^-ATPase-containing vesicle in alveolar epithelial cells [20]. We further analyzed the expression patterns of Na^+^/K^+^-ATPase in the kidneys of adult mice by double labelling of Na^+^/K^+^-ATPase and of markers of specific nephron segments (Fig 5). It was found that Na^+^/K^+^-ATPase was basolaterally localized in the epithelial cells of THP-positive TAL and NCC-positive DCT (Fig 5D–5I), but not expressed in the LTL-positive PT or DAB (Dolichos biflorus agglutinin) positive CD (Fig 5A–5C and 5J–5L). Moreover, Kif5b and Na^+^/K^+^-ATPase were found to be co-localized in the same tubular structures (Fig 5M–5O), suggesting that Kif5b performs regulatory functions in Na^+^/K^+^-ATPase-containing vesicle transportation in renal epithelial cells of TAL and DCT that are similar to the functions that it performs in alveolar epithelial cells.

Basolaterally localized Na^+^/K^+^-ATPase can move sodium from the cell into the blood by consuming ATP, so as to establish and maintain an electrochemical gradient within the cell. Since Kif5b has been reported to transport mitochondria within cells [9, 32], the basolateral distribution of Kif5b may facilitate the translocation of mitochondria to the vicinity of Na^+^/K^+^-ATPase where there is a high demand for ATP to sustain the transport of sodium out of the cell. In addition to Na^+^/K^+^-ATPase, there are also other transporters/receptors located on the basolateral membrane in TAL and DCT, which regulate sodium, calcium, and potassium re-absorption. By sensing the interstitial divalent mineral ion concentration, the basolaterally-localized Ca^2+^-sensing receptor (CaSR) [33] can regulate the transport efficiency of apical transporters: the Na-K-2Cl symporter (NKCC2) and K^+^ channel in TAL, and the Na-Cl symporter (NCCT) in DCT [34]. The basolateral distribution of insulin-dependent (GLUT4) and insulin-independent glucose transporters (GLUT1) in the epithelial cells of TAL is important for electrolyte transport [35, 36]; and in adipocytes, insulin-stimulated GLUT4 translocation is known to be mediated by Kif5b [15]. The involvement of Kif5b in the basolateral targeting of these receptors and/or transporters awaits further investigation. Nevertheless, it is possible that a deficiency of Kif5b in these tubules may make the re-absorption of sodium, potassium and chloride less efficient, thereby leading to renal disease similar to Bartter syndrome and/or Gitelman syndrome, which are electrolytic metabolic disturbance syndromes resulting from genetic mutations of ion channels such as NKCC2 in the TAL and NCCT in the DCT [37].

### Kif5b knock-down kidney shows normal histological morphology and reduced cell proliferation

**Fig 6:**
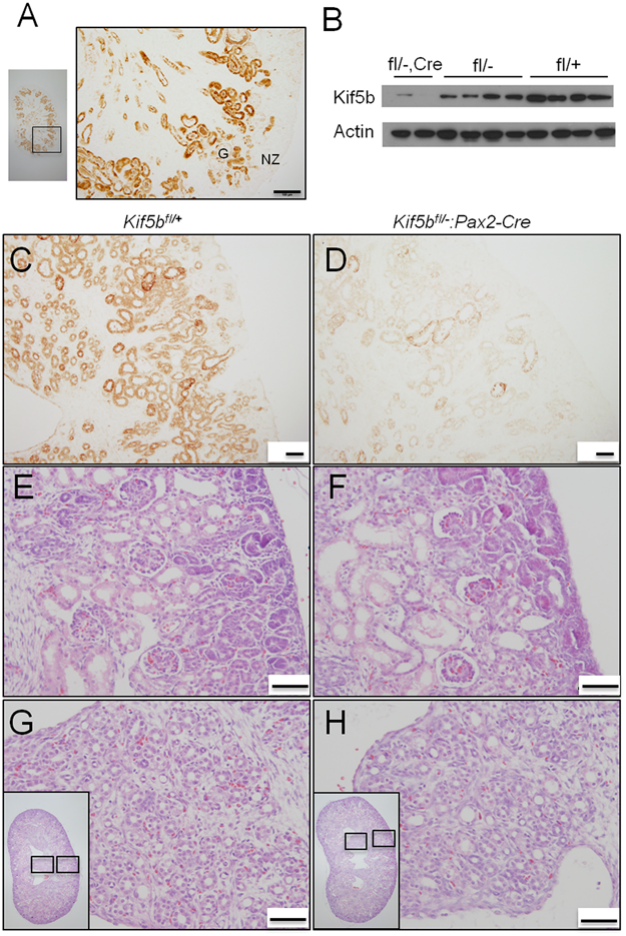
Kidneys from Kif5b-Pax2KD (*Kif5bfl/-*:*Pax2-Cre*) embryos show normal gross histological morphology. (A) Anti-β-galactosidase immunostaining of E19.5 *R26R* ^*+/+*^:*Pax2-Cre* mouse kidney. (B) Western blot analysis of Kif5b levels in kidneys from E19.5 mutant (*Kif5b* ^*fl/*^ *-*:*Pax2-Cre*), heterozygous (*Kif5b* ^*fl/-*^ and *Kif5b* ^*fl/+*^:*Pax2-Cre*) and wild-type (*Kif5b* ^*fl/+*^) embryos. Actin was used as a loading control. (C-D) Immunohistochemical analysis of Kif5b expression patterns/levels in kidneys from E19.5 control (*Kif5b* ^*fl/+*^) and mutant (*Kif5b* ^*fl/-*^:*Pax2-Cre*) embryos. (E-H) H&E staining showing that the organization of the nephrogenic zone, cortex and medulla is similar between control (*Kif5b* ^*fl/+*^) and mutant (*Kif5b* ^*fl/-*^:*Pax2-Cre*) kidneys. G, glomerulus; NZ, nephrogenic zone. Images are representative of kidney tissue sections from ten E19.5 embryos. Scale bar = 50 μm.

In order to analyze the function of Kif5b in renal epithelial cells, conditional knock-down mice Kif5b-Pax2KD (*Kif5b* ^*fl/-*^:*Pax2-Cre*) were generated by crossing *Kif5b* ^*+/-*^:*Pax2-Cre* mice with *Kif5b* ^*fl/fl*^ mice. Kif5b-Pax2KD mice died with limb muscle dystrophy at the perinatal stage, probably due to hypoxia and/or impairment of milk intake [23]. The Pax2-Cre activity has been proven to efficiently delete LoxP-flanked sequences in Pax2-expressing cells and their descendants [23, 38]. Our analysis showed that positive immunostaining of β-galactosidase was observed in the ureteric bud and developing renal tubules of the kidneys of E19.5 *R26R* ^*+/+*^:*Pax2-Cre* embryos (Fig 6A), which overlapped with the location of expression of Kif5b. A decrease of approximately 90% in the level of Kif5b was observed in the kidneys of E16.5 [23] and E19.5 Kif5b-Pax2KD embryos (Fig 6B–6D). The overall histological morphology of kidneys was normal in E19.5 Kif5b-Pax2KD embryos (Fig 6E–6H). The three-layer arrangement of nephrogenic zone, cortex and medulla appeared to be as well organized in the mutant kidneys as in control kidneys. Thus, in the kidneys of the mutant mice, the cortex comprised both glomeruli and developing tubules (Fig 6E and 6F); and furthermore, the overall cellularity, asymmetry, size of the glomeruli in the mutant kidneys were comparable to those in control kidneys (Fig 6G and 6H). In the medullas of both groups of kidneys, the collecting ducts and loops of Henle formed rays in parallel from the cortex to the deep medulla, with the interstitial cells aligned perpendicularly to support these ducts (Fig 6G and 6H). No dilation or cyst formation was found (Fig 6E–6H). Taken together, no obvious abnormality in the morphogenesis of the glomeruli or the renal tubules could be detected by histological examination.

**Fig 7:**
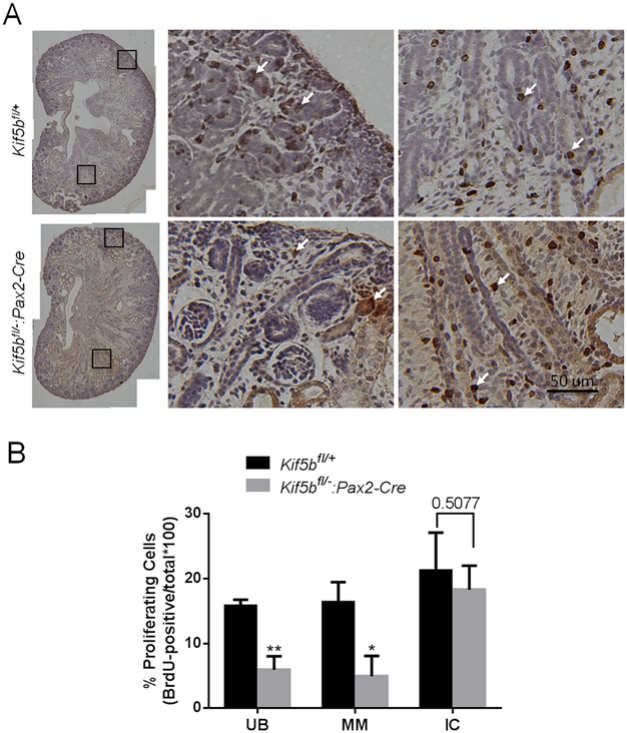
Kidneys from Kif5b-Pax2KD (*Kif5bfl/-*:*Pax2-Cre*) embryos have a reduced cell proliferation rate. (A) *In situ* analysis of BrdU incorporation at E18.5. Pregnant mice were injected with BrdU and sacrificed 4 hours later. BrdU incorporation was detected by an *in situ* BrdU incorporation assay kit. Brown-stained nuclei are BrdU-positive. Tissues were counterstained with hematoxylin. Images are representative of kidney tissue sections from three embryos. Arrows, BrdU positive cells. Scale bar = 100 μm. (B) Quantitative analysis of BrdU incorporation at E18.5. BrdU incorporation is expressed as the percentage of the total number of cells in the ureteric bud and its branches (UB), in the metanephric mesenchyme (MM) or in the interstitial cells (IC). *Kif5b* deficiency significantly decreased BrdU incorporation in both ureteric bud and mesenchyme-derived epithelial structures, compared to wild type. Bars represent the means ± S.D. Six sections from three wild type and three mutant embryos were imaged and counted. *P* values are indicated in the figure. * *P*<0.05.

Dysregulation of cell proliferation and/or apoptosis results in renal dysplasia [39]. We examined the impact on cell proliferation of abolishing Kif5b-mediated intracellular transportation using BrdU labeling. As shown in Fig 7, BrdU incorporation was observed in metanephric mesenchyme cells, mesenchyme-derived epithelial structures and ureteric buds in the outer regions of kidneys, as well as interstitial cells located in the medulla region (Fig 7A). Quantitation of the number of BrdU^+^ cells in ureteric bud branches (UB) and in metanephric mesenchyme (MM) revealed an approximately two- to three-fold diminution of cell proliferation in the kidneys of Kif5b-Pax2KD mice. By contrast, the cell proliferation rate was not affected in interstitial cells (IC) (Fig 7B). Based on TUNEL^+^ analysis, there was no detectable increase in renal cell apoptotic rate (S2 Fig).

In mice, pronephros (the predecessor of kidney) appears at E7.5 and develops to functional mesonephros at E9.5 and metanephros at E11 [2, 3, 40]. Afterwards, active nephrogenesis occurs to generate the mature kidney. The exact time at which *Kif5b* is knocked out in the kidneys of Kif5b-Pax2KD mice needs to be verified. However, Pax2-Cre starts to express at E9.5 [38], suggesting that *Kif5b* may be deleted as early as E9.5. Furthermore, expression of Kif5c mRNA in E12.5 kidneys (Fig 2) suggests that Kif5c may have a redundant function in replicating the role of Kif5b during kidney embryonic development and that this may contribute to the lack of obvious morphological abnormality in the kidneys of E19.5 mutant embryos.

In this study, we found that kidney-specific knock-down of *Kif5b* resulted in reduced cell proliferation rate in UB and MM. Similar phenotype was observed in *Kif5b* depleted pancreatic β cells, which resulted in reduced islet size in mutant mice [24]. Kif5b depleted Hela cell as well as MCF-7 cell also displayed growth inhibition [39]. *Kif5b* null mice showed midgestation lethality with severe growth retardation during 9.5–11.5 dpc [9]. *Kif5b* null embryos can survive the first several embryonic days indicating that cells can proliferate without Kif5b in specific cell types, but the proliferation rate was substantially decreased. However, primary cultures of *Kif5b* null myoblast cells had normal growth rate and cell cycle distribution pattern, indicating that Kif5b is dispensable for myoblast cell proliferation [23]. The specific function of Kif5b in cell proliferation is unclear up to now and it deserved further investigation.

### Mislocalization of Na^+^/K^+^-ATPase in the kidneys of Kif5b knock-down embryos

**Fig 8:**
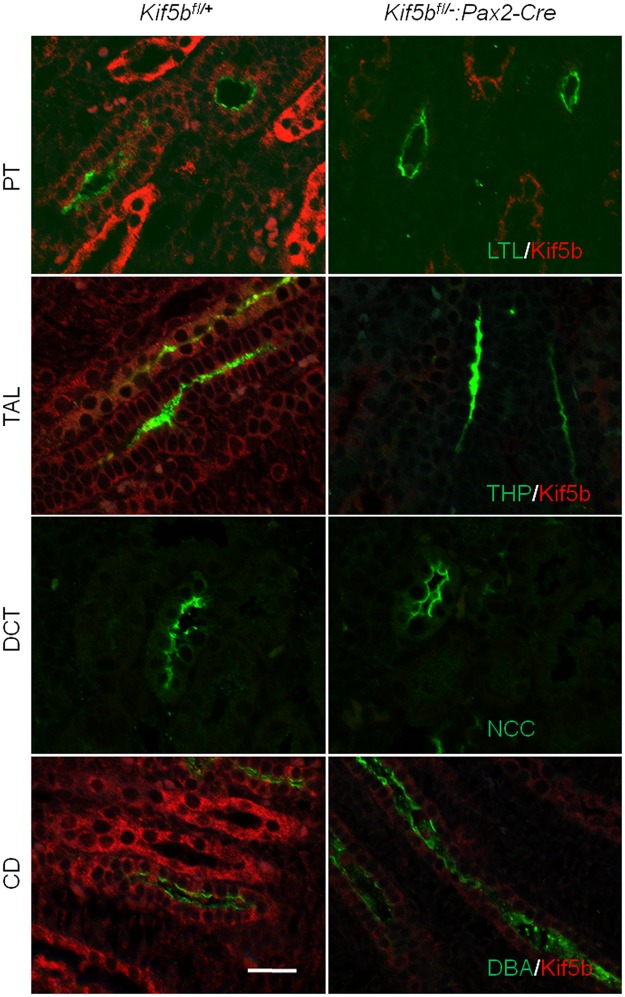
Kif5b deficient kidneys (*Kif5bfl/-*:*Pax2-Cre*) show normal localization of apical markers. Co-immunostaining of Kif5b (red) and apical markers LTL, THP, NCC and DBA (green) in kidney sections of E18.5 embryos. Images are representative of kidney tissue sections from three mice. Scale bar = 25 μm.

**Fig 9:**
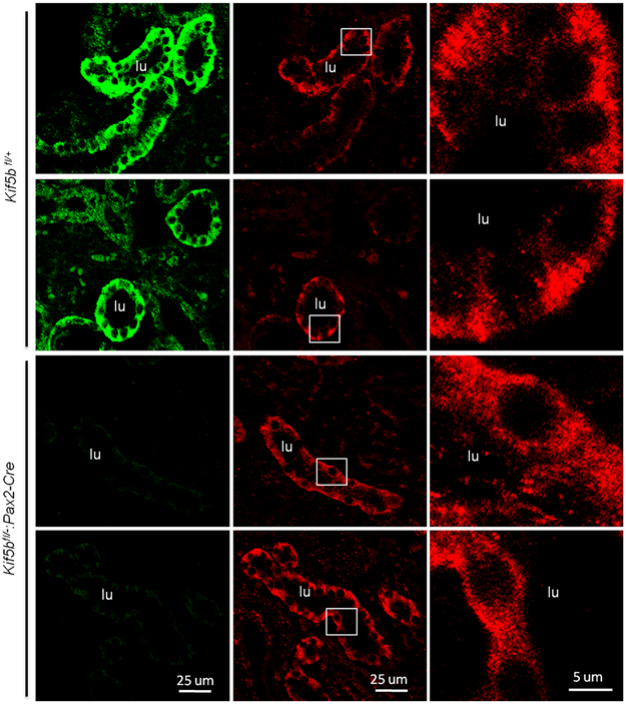
Mislocalization of Na^+^/K^+^-ATPase in Kif5b knock-down kidneys. Na^+^/K^+^-ATPase was basolaterally localized in renal epithelial cells of control E18.5 embryos (*Kif5b* ^*fl/+*^), but it was mislocalized to the apical surface in renal epithelial cells of mutant E18.5 embryos (*Kif5b* ^*fl/-*^:*Pax2-Cre*). Images are representative of kidney tissue sections from three mice. Green, Kif5b; Red, Na^+^/K^+^-ATPase.

The tubular localization of Kif5b in un-weaned mice, and the selective and asymmetric intracellular distribution of Kif5b both in the TAL and in the DCT in weaned mice, suggests that Kif5b is involved in physiological functions in renal tubules. Unfortunately, the early death of Kif5b-Pax2KD mice makes it impossible to undertake a functional analysis of Kif5b in the kidney. We analyzed the polarity of tubules by staining tissue sections with polarity markers (Figs 8 and 9). LTL, THP, NCC and DBA are apical markers of PT, TAL, DCT and CD, respectively. As in the kidneys of *Kif5b* ^*fl/+*^ mice, all these markers were localized in the apical region of tubular epithelial cells in the mutant kidneys, indicating that the apical localizations of LTL, THP, NCC and DBA were not affected following the knock-down of Kif5b (Fig 8). However, the basolateral marker Na^+^/K^+^-ATPase was found to be present on the apical surface of tubular epithelial cells following the knock-down of Kif5b (Fig 9), suggesting that Kif5b is the motor protein involved in the transportation of Na^+^/K^+^-ATPase-containing vesicles in renal epithelial cells.

Trejo *et al*. have previously reported that the traffic of the Na^+^/K^+^-ATPase depends on functional Kinesin-1 in the alveolar epithelium [20]. Here we provide direct evidence for the role of Kinesin-1 in the traffic of Na^+^/K^+^-ATPase in mouse kidney. We found that Kif5b co-localized with Na^+^/K^+^-ATPase, and knock-down of Kif5b depolarized Na^+^/K^+^-ATPase but did not affect the polarity of apical proteins. Since the Na^+^/K^+^-ATPase generates the electrochemical gradient that drives the movement of sodium from the cell into the blood [29], it could be suspected that genetic or epigenetic alteration in Kif5b (or Kinesin-1) may result in renal failure through depolarization of Na^+^/K^+^-ATPase,

The physiological importance of Kif5b in mouse kidney is not clear and it remains uncertain whether Kif5b in the kidney is essential for mouse survival. In order to answer these questions, a more specific conditional knockout mouse model needs to be developed.

## Supporting Information

S1 Fig (A) Amino acid sequence alignment for Kif5 member, 5-a, -b, -c. High variable sequences (underlined) in the neck region were selected as the antigens for Kif5b and Kif5c. Sequences (NGNATDINDNRSDLPC (992-1006aa)) located at the tail domain of Kif5a was selected as the antigen for Kif5a. (B) Purificaiton and testing the specificities of anti-Kif5a, 5b and 5c antibodies by western blot using mouse brain tissue lysate as the input. It is demonstrated that the anti-Kif5a (1:100), anti-Kif5b (1:1000) and anti-Kif5c (1:100) recognize protein bands of about 130, 120 and 110–120 kD respectively. Specific peptides can block the signals from the above antibodies in the Western blot. (C) The specificity for anti-Kif5b antibody has also validated on kidney tissue sections by immunostaining. Kif5b was strongly expressed in all developing tubules and ureteric bud in P0 kdineys (S1C Fig, upper left) and basolateral domain of some renal tubues in mature kidneys (S1C Fig, upper right). The positive staining was absent after preincubating the anti-Kif5b antibody with the 18-aa synthesized peptides (S1C Fig, bottom). Images are representative of kidney tissue sections from three mice. Scale bar = 50 μm. (D) Kif5a and Kif5c were expressed in the brain but not in the kidney of new born mice. (E) Western Blot analysis of Kif5b expression levels at various postnatal developmental stages. Kif5b protein level in the whole kidney was constant during mouse kidney postnatal development.
(TIF)

S2 Fig Representative photo of TUNEL assay in sections from positive control (CT), wild type mice (*Kif5b* ^*fl/+*^) and mutant mice (*Kif5b* ^*fl/-*^,*Cre*). Images are representative of kidney tissue sections from three mice. Scale bar = 25 μm.
(TIF)

S1 Table Primer pairs used in real-time PCR. (DOCX)
