# Supplementary figures and images for: Analysis of Kif5b Expression during Mouse Kidney Development
Source: PLoS One. 2015 Apr 17;10(4):e0126002. doi: 10.1371/journal.pone.0126002 (PMC4401754; doi:10.1371/journal.pone.0126002)

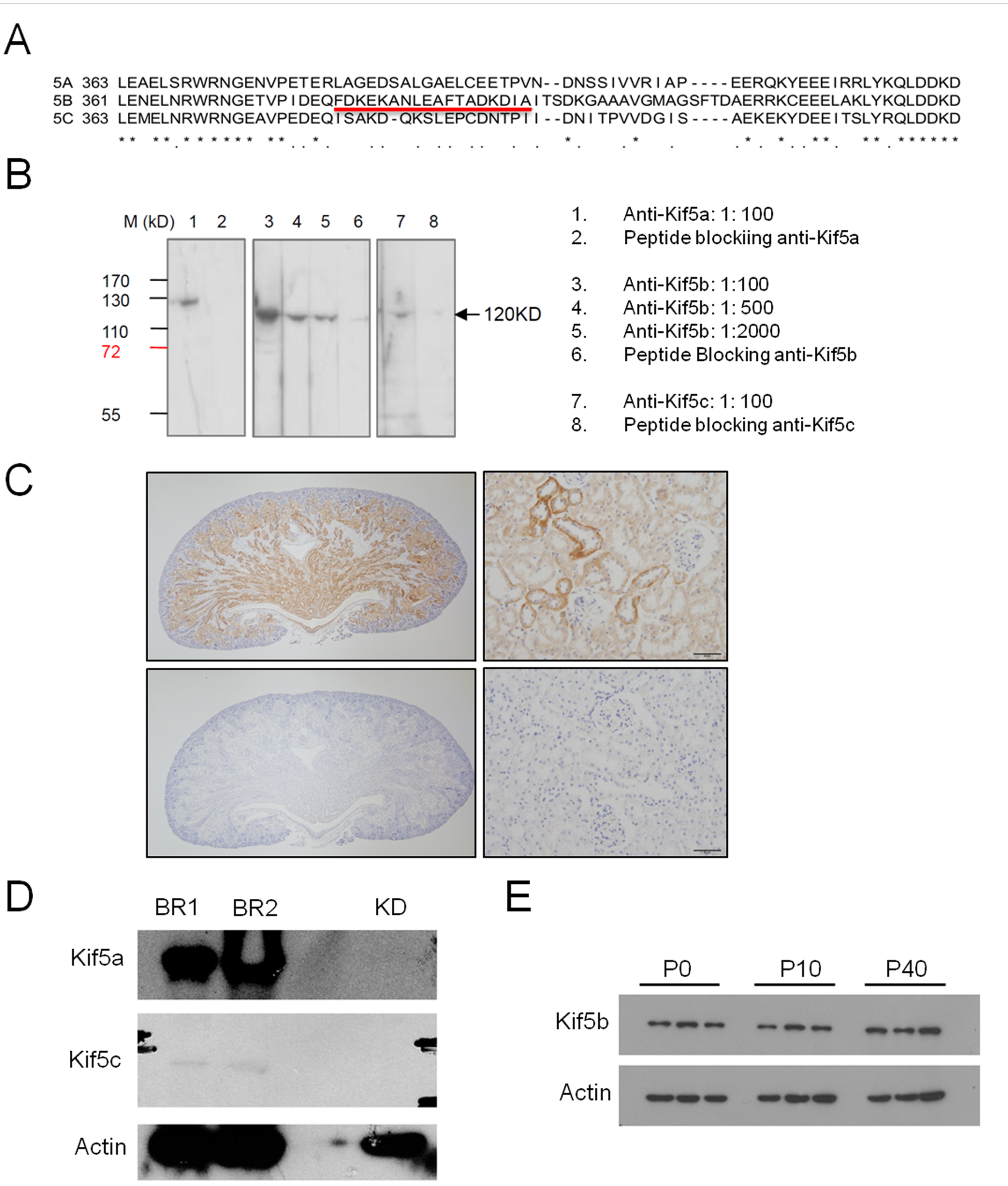

Supplement: S1 Fig — (A) Amino acid sequence alignment for Kif5 member, 5-a, -b, -c. High variable sequences (underlined) in the neck region were selected as the antigens for Kif5b and Kif5c. Sequences (NGNATDINDNRSDLPC (992-1006aa)) located at the tail domain of Kif5a was selected as the antigen for Kif5a. (B) Purificaiton and testing the specificities of anti-Kif5a, 5b and 5c antibodies by western blot using mouse brain tissue lysate as the input. It is demonstrated that the anti-Kif5a (1:100), anti-Kif5b (1:1000) and anti-Kif5c (1:100) recognize protein bands of about 130, 120 and 110–120 kD respectively. Specific peptides can block the signals from the above antibodies in the Western blot. (C) The specificity for anti-Kif5b antibody has also validated on kidney tissue sections by immunostaining. Kif5b was strongly expressed in all developing tubules and ureteric bud in P0 kdineys (S1C Fig, upper left) and basolateral domain of some renal tubues in mature kidneys (S1C Fig, upper right). The positive staining was absent after preincubating the anti-Kif5b antibody with the 18-aa synthesized peptides (S1C Fig, bottom). Images are representative of kidney tissue sections from three mice. Scale bar = 50 μm. (D) Kif5a and Kif5c were expressed in the brain but not in the kidney of new born mice. (E) Western Blot analysis of Kif5b expression levels at various postnatal developmental stages. Kif5b protein level in the whole kidney was constant during mouse kidney postnatal development. (TIF) [file pone.0126002.s001.tif]

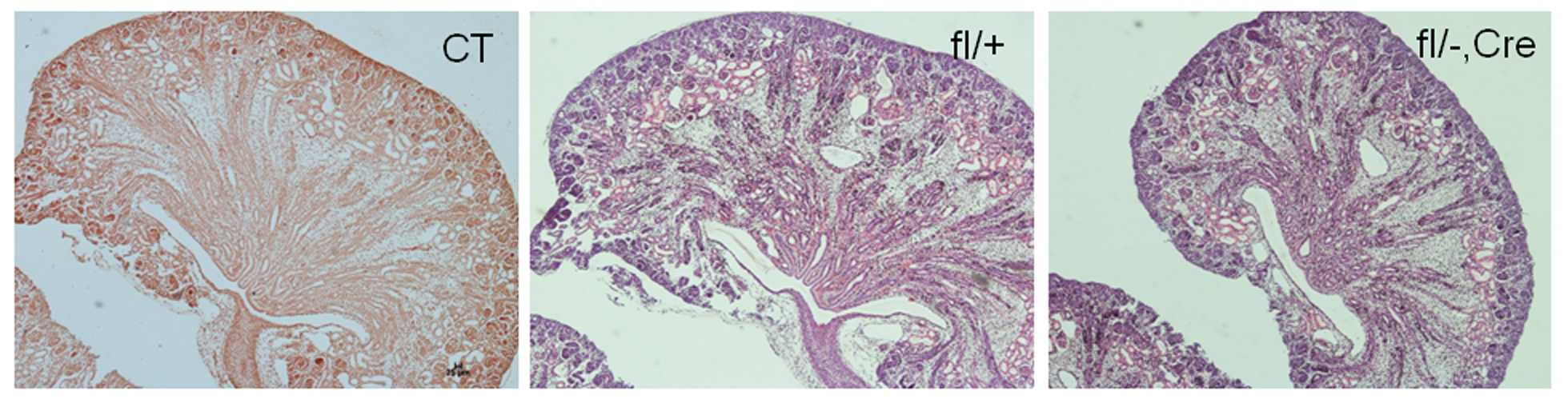

Supplement: S2 Fig — Images are representative of kidney tissue sections from three mice. Scale bar = 25 μm. (TIF) [file pone.0126002.s002.tif]
