# Supplementary material for: Analysis of Kif5b Expression during Mouse Kidney Development
Source: PLoS One. 2015 Apr 17;10(4):e0126002. doi: 10.1371/journal.pone.0126002 (PMC4401754; doi:10.1371/journal.pone.0126002)
Supplement: S1 Table — (DOCX) [file pone.0126002.s003.docx]

**S1 Table. Primer pairs used in real-time PCR**

| Gene name | Accession no.(NCBI) | Forward primer  5’-3’ | Reverse primer  5’-3’ |
| --- | --- | --- | --- |
| *Kif5a,V1* | NM_008448.3 | CCAGATCGCTAAGCCTGTGA | TGGGGAGGGAGTTAGTGTCA |
| *Kif5a, V2* | NM_008447.4 | ACATGGACAATGGAAATGCCAC | CCTGCAGCTACCTGAAAGTGC |
| *Kif5b* | NM_008448.3 | CCAGATTGCAAAGCCGATCC | GGAGCAGAGTCCCTCAACAC |
| *Kif5c* | NM_008449.2 | CATTCGGCTCAGATCGCCAA | CTCCAGAGACCACACAACCC |
| *18S RNA* | NR_003278 | GTAACCCGTTGAACCCCATT | CCATCCAATCGGTAGTAGCG |

.
